# CBCT Evaluation of Quality and Quantity of Bones for Immediate Implant Treatment Planning in Central Incisor Area in relation to Arch Form

**DOI:** 10.1155/2023/8863318

**Published:** 2023-04-17

**Authors:** Nuhad A. Hassan, Afya Sahib Diab Al-radha

**Affiliations:** ^1^Oral Medicine Department, College of Dentistry, Al-Mustansiriyah University, Baghdad, Iraq; ^2^Oral Surgery and Periodontology Department, College of Dentistry, Mustansiriyah University, Baghdad, Iraq

## Abstract

Understanding the quality of the ridge and facial cortical bone in the aesthetic zone is important for treatment with an immediate dental implant. This study aimed to analyze bone density and widths of the facial cortical bone and alveolar ridge at the central incisors in relation to arch form. A total of 400 teeth from 100 cone-beam CT images were divided equally between the upper and lower central incisors. The central incisor area was assessed for the width of the facial cortical and alveolar bones at three different points (3 mm, 6 mm, and 9 mm from the cementoenamel junction). Arch forms and densities of cortical and cancellous bones in the interradicular regions were evaluated. The difference in facial cortical bone thickness at 3 points was smaller for the upper teeth than for the lower teeth on both sides. The alveolar bone width was higher in the maxilla than the mandible with highly significant differences (*P* < 0.001). The highest bone density was at the buccal aspect of the mandible (897.36 ± 136.72 HU), while the lowest density was at the cancellous bone of the maxilla (600.37 ± 126.63 HU). The dominant arch form was ovoid 71%, followed by square 20% and the tapering arch form 10%. The tapering arch form has the highest alveolar bone width in the upper jaw without statistical significance. The facial cortical bone thickness needs to be evaluated before implantation in the anterior region because it is less than two millimeters in both jaws. CBCT is important for the immediate implant. The ovoid shape was the dominant arch form.

## 1. Introduction

Dental implants have become an important option in the treatment plan in dentistry. However, with the great advanced technique and technology of implantology, several approaches for implant insertion, placement, and treatment planning had been developed [1–4]. The placement of an immediate implant in the anterior region is one of these developed strategies and had gained an acceptable reputation [5].

One of the problems usually faced by implantologists is the deficiency in the necessary bone required to support the dental implant, particularly the buccal bone for immediate implant in anterior teeth, which usually required bone augmentation to compensate for such a defect in the buccal wall [6].

Bone quality and quantity are necessary for a successful implant placement in the upper and lower jaws. However, “con-beam computed tomography” (CBCT) has been widely used for the surgical planning in dental implant treatment, assessment of bone around dental implant, and evaluation of bone “microstructure,” and the quantity of bone in both jaws can be evaluated by CBCT through implant treatment [7–9].

However, CBCT can be used to evaluate and measure the facial cortical bone thickness (FCT) at various levels along the root in pre- or postextraction [10] and also can be used for analyzing the available bone in case of quantity and shape that is required for the prospected implant site [11].

The anterior maxilla area is considered a difficult area to restore with dental implant treatment and presents more challenges because well-anchored implants and aesthetic outcomes were critical in such an area. It also required great attention for the preoperative assessment of the available bone quantity and quality to ensure good aesthetic and functional results after implant placement [12].

Arch form is one of the fundamental components that need to be determined before deciding the dental implant treatment plane, as the number of implants required and the type and shape of prosthesis differ between different arch forms [13] The simplest classification to categorize dental arch form types is “square, ovoid, and tapering” [13].

This study aims to examine the variations in bone density and widths of the facial cortical bone and alveolar ridge at the central incisor area and whether there is an association between these variables and the type of arch form.

## 2. Materials and Methods

A total of 400 teeth from 100 CBCT images were divided equally between the upper and lower central incisors. Participants' ages ranged from 20 to 50 years. CBCT scans were performed to diagnose dental conditions. All scans were performed by “Kodak 9500, Care Stream, France; 10.8 s exposure time; 10 mA; 90 KV; voxel size of 300 *µ*m.”

Exclusion criteria: anterior teeth with periapical pathology, periodontal disease, root canals, restoration, crowns, or dental implants were not included in this study. However, for the subsample which evaluates the arch form, all the anterior maxillary teeth must exist without any crowding or spacing.

Data collection took place between March 2021 and December 2021. It was ethically approved by the Scientific Committee of the Department of Oral Medicine in accordance with the “World Medical Association and the Declaration of Helsinki” (Protocol 14-2-2021).

Using “G^*∗*^Power 3.0.10 (program written by Franz-Faul, University of Kiel, Germany), with the power of study = 85%,” after conducting a pilot study for 10 teeth in each jaw, alpha error of probability = 0.05, the effect size of bone density between the upper and lower jaws is 0.3 (weak). Under all these conditions the sample size is 200 teeth in each jaw (400 teeth total).

A subsample was taken to evaluate the association of bone measurement variables with arch form; in this subsample, all the anterior maxillary teeth must exist without any crowding, spacing, restoration, crowns, or dental implants.

The linear measurements of the anterior maxilla described by Misch were used to determine the dental arch form “Misch's classification.” In [13], the measurements were conducted using the measuring tool in the CBCT view according to Somvasoontra et al.'s measurement [14].

**Figure 1:**
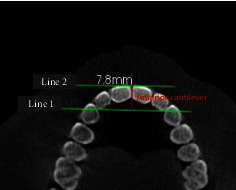
Representative images showing the linear measurements of the anterior maxilla that are used to determine dental arch forms according to “Misch's classification.” The first line connected between “canine cusp tips”; the second line was drawn that touched the most prominent area of the facial surface of the upper central incisor and was parallel to the first line in the axial plane. The distance between these two imaginary lines determines the type of arch form.

In brief, after adjusting the axial, sagittal, and coronal planes, the canine's cusp tips were determined in the axial plane and the first line that connected between “canine cusp tips” was determined; then, a second line was drawn which touched the most prominent area of the facial surface of the upper central incisor and parallel to the first line in the axial plane, as shown in Figure 1. The type of arch form will be determined according to the distance between these two imaginary lines (square < 8 mm; ovoid 8−12 mm; tapering > 12 mm) [13, 14].

Measurements were made by one “radiologist” (N.H.A.), and they were repeated by the same “radiologist” after five days. Means were taken for each parameter.

A paired *t*-test was conducted on 10 randomly chosen CBCT measurements to demonstrate intraobserver reliability, and the results showed no statistically significant differences between the two readings.

**Figure 2:**
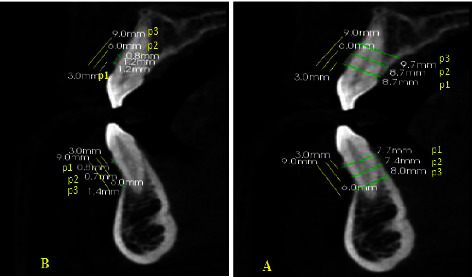
Representative images showing (a) the measurements of alveolar bone width. (b) Measurements of facial cortical bone width both measured for the upper and lower jaws at three points ((P1: 3 mm; P2: 6 mm; P3: 9 mm) from the cementoenamel junction).

As shown in Figure 2, both central incisors of the upper and lower jaws were assessed for the width of facial cortical bone (FCT) and alveolar bone width (AW) at three different points: P1 (3 mm), P2 (6 mm), and P3 (9 mm) from the cementoenamel junction (CEJ). At each point, the estimation was made perpendicular to the tooth axis. In each jaw, both the right and left central incisors were included for each participant [15]. The densities of cortical and cancellous bones were measured 5 mm from the root apex of the coronal and interradicular regionsbetween the two central regions. The midpoint of the cortical bone was selected for measuring density. In the cancellous bone, the half distance between bucco-lingual and palatal bones was used for measurement [16]. Hounsfield units were recorded using Kodak software.

Statistical analysis was conducted using “Excel” and “IBM SPSS Statistics 24 programs.” The results assessed were descriptive statistics: mean and standard deviation.

After verification of the “normal distribution and homogeneity of variance,” a *t*-test was used to assess the significant differences between the groups. Statistical analysis (independent *t*-test) was performed to test the difference in bone width between the upper and lower groups and between the left and right sides at each of the three points and in different arch forms. “The probability value (*P* value) is considered significant at *P* < 0.05 and highly significant if *P* < 0.01.”

## 3. Results

Alveolar and facial cortical bone widths of 400 teeth from 100 participants were estimated for the central incisors of both the upper and lower jaws at three points.

Arch form results were obtained on a subgroup from the data where only 82 of the participants were included, as 18 patients were excluded, because the arch form measurement was not conducted on their CBCT (7 have impacted canine; 4 have missed canine; 7 have rotated malposition canine).

The dominant arch form was ovoid 71% (*n* = 58), followed by the square arch form at 28.4% (*n* = 23) and the lowest percentage was the tapering at 10% (*n* = 8).

**Figure 3:**
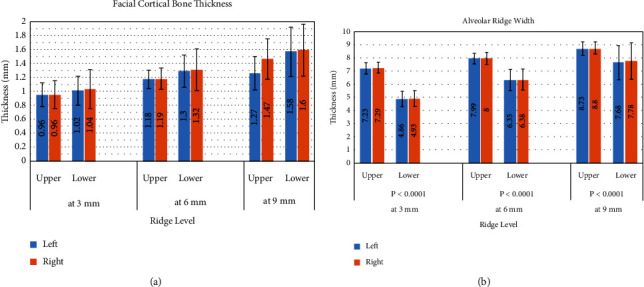
Bone width in millimeters of the upper and lower central incisors (both sides) at 3 levels (3 mm, 6 mm, and 9 mm from the cementoenamel junction) (*n* = 100 for each). Error bars represent the standard deviations. *P* values in *X*-axis represent the significance between the upper and lower measurements at the same level. (a) Facial cortical bone thickness; (b) alveolar bone width.

Figure 3(a) shows the means and standard deviation (SD) for facial cortical bone thickness (FCT) of the upper and lower jaws for each side; it shows the steady increase in bone thickness from the crest (P1) to apical parts for upper and lower incisors. The mean of FCT measurements was slightly more in the mandible than in the maxilla with no significant difference between the upper and lower measurements, nor between the left and right measurements.

However, the opposite picture is shown in Figure 3(b), where the alveolar bone width (AW) is remarkably greater in the upper compared to the lower with highly significant differences at 3 points and for both sides (*P* < 0.001).

**Figure 4:**
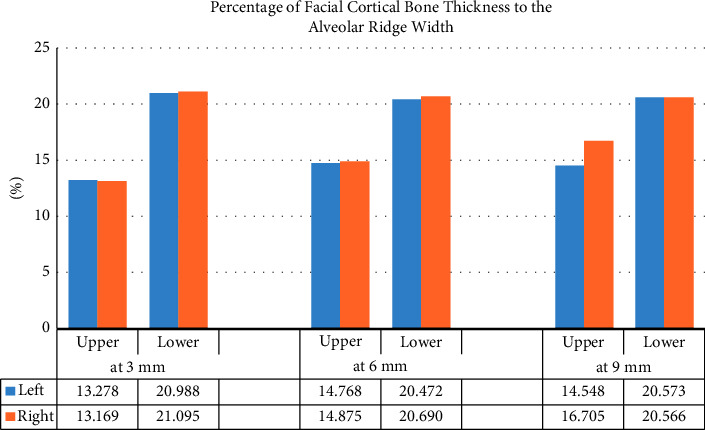
Percentage (%) of facial cortical bone width to alveolar bone width for the upper and lower central incisors (both sides) at 3 levels.

One of the noticeable findings is the higher percentage of facial cortical bone width to the alveolar ridge width in lower incisors than in the upper incisors, which is shown in Figure 4.

Table 1 shows a significant difference between the upper and lower jaws in the bone density and among facial and palatal/lingual aspects of both jaws. In the case of cortex densities, the mandibular facial cortex recorded the largest density (897.36 ± 136.72 HU), while the lowest cortical bone density was at the facial cortex of the maxilla (720.75 ± 103.17 HU). A significant difference was noted between the facial and palatal/lingual cortical bone densities of both jaws (*P*=0.039 for the upper and *P*=0.046 for the lower). Additionally, the difference was significant between the upper and lower jaws among facial cortical densities (*P* ≤ 001) and between the palatal and lingual cortical densities (*P*=0.034) as well as between the upper and lower cancellous bone densities (*P* ≤ 001).

**Figure 5:**
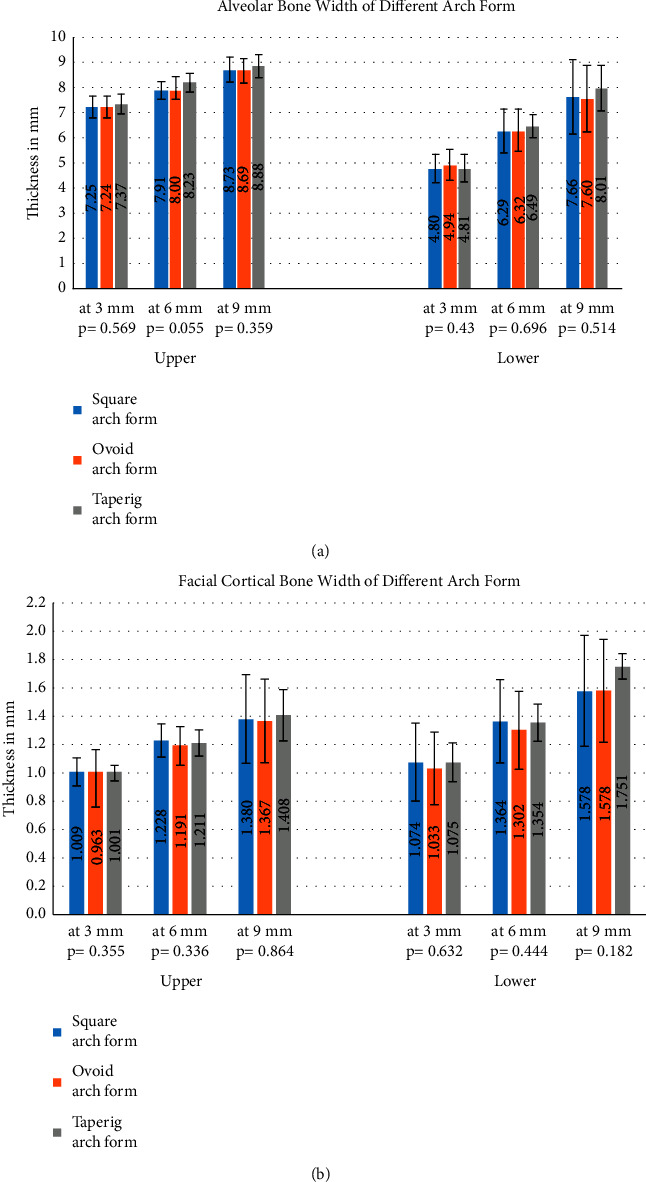
Bone width in millimeters of the upper and lower central incisors (both sides) at 3 levels according to the arch form type. Error bars represent the standard deviations. *P* values represent the significance between the three-level point measurements in the same arch form group type. *P* values on the *X*-axis represent the significance between alveolar bone width measurements at the same level and the same arch. (a) Alveolar bone width and (b) facial cortical bone width.

In the case of the arch form, AW had a similar picture for all arch forms in which the AW increased steadily from point P1 to point P3 (Figure 5(a)). The tapering arch form had the highest alveolar bone width in the upper jaw in three-point measurements without statistical significance, while in case of the lower jaw, the tapering arch form had the highest AW in point P2 and point P3 and also without any statistical significance.

The captured information from Figure 5(b) is that the ovoid arch form has the lowest FCT measurement in both the upper and lower jaws and the three-point measurements, but again without any significance.

However, a detailed measurement regarding FCT and AW in both upper and lower arches according to arch form type and gender is shown in Table 2. Both the FCT and AW were greater in the male than in the female in three points of measurement and all arch form types, as shown in Table 2.

## 4. Discussion

The central incisor area was assessed for facial cortical and alveolar bone widths at three different points in relation to arch form. Cortical and cancellous bone densities in the interradicular regions were also evaluated. The alveolar bone width was higher in the maxilla than in the mandible with the tapering arch form having the highest alveolar bone width in the upper jaw, while the facial cortical bone thickness was smaller for the upper than the lower teeth on both sides.

Cone-beam CT is a three-dimensional imaging tool, which is one of the most advanced methods available for examining the bone. It can be used as a precise and accurate tool to assess buccal bone height and thickness [17].

The thickness of cortical bone has often been studied using advanced imaging in the literature; however, few studies have differentiated between sites or dealt with the mandible [18]. Therefore, in this study, measurements of AW and FCT of the upper and lower central regions were made by CBCT.

This work aligns with most prior studies that have explored the mandibular bone thickness on the facial side [19]. Also, López-Jarana et al. [20] estimated the thickness of the facial wall at three different points on the upper and lower incisors in 49 CBCTs. Both the upper and lower incisors showed a high percentage of thickness less than ideal, confirming CBCT's valuable role in implant treatment following extraction; indeed, current findings also support this role.

Based on the existing study, the upper and lower FCT were 1.47 ± 0.299 and 1.66 ± 0.373, respectively, which are consistent with Zekry et al. [10] who found that very rarely >2 mm thickness was present within 200 images of each jaw.

The current study results indicated that the lower facial cortical bone was larger than the upper. Also, the width tended to increase as far from the crest towards the apex; this finding is inconsistent with the previous studies [21, 22]. However, in the current study, the thickness of the buccal cortex of the mandible is higher than the maxillary which is in line with both Park and Cho and Farnsworth et al.'s findings [23, 24]. On the contrary, Katranji et al. [25] found a large variation of the buccal plate thickness across the maxilla and mandible from 1.6 to 2.2, the thinnest being in the lower anterior region. This indicates that these differences may arise from different measuring segments in each jaw and on cadavers.

However, according to Chen et al. [26], the anterior maxilla had a thin bone thickness of less than 1.5 mm; their finding was consistent with this study. This may implicate considerable rates of problems within implants, which necessitate the use of CBCT in the early stages of implantation to identify anatomical characteristics that may adversely affect placement [27].

As a baseline, FCT affects postextraction morphological alterations of the alveolar bone; sites with more than one millimeter of FCT experienced smaller resorption than the ridge with one millimeter of FCT [28]. Therefore, a detailed review of facial cortical bone structure and thickness is imperative as it will influence the decision for an immediate implantation process [29].

Based on the study findings, the alveolar ridge width was thicker in the upper than lower. This could be related to the increase in cancellous bone trabeculae in order to compensate for the thin upper facial cortex. Also, the difference in teeth size between the upper and lower could explain such a difference.

As previously discussed in the literature, the cortex width is more important for dental implant stability in the initial healing periods after implant placement than the implant fixture's length, and the ratio of cancellous to cortical bones is also crucial in the implant placement area [30]. One of the interesting results is the higher percentage of facial FCT to the alveolar ridge width in the lower incisor area than in the upper. Nevertheless, in close examination of the results, one can notice that relatively there is no difference in FCT between the upper and lower teeth, and this difference in the ratio happened because of the significant difference in the AW that led to this remarkable difference in ratio results.

It has been reported that bone thickness around incisors in the cervical area is lower than the thickness in the apical area in normally aligned maxillary central incisors [31–33]. These results differ when examining teeth that have buccal or palatal/lingual inclination as the apical bone thickness decreased when the teeth have Lingual-inclination [32] and increased when the teeth have a buccal inclination [34] with positive correlation with the angle between the long axis of the upper central incisor and the palatal plane [31]. However, despite Tian et al. finding similar results, they stated that the midroot areas remain with very slight differences in bone thickness with little change between different angulations in the labiolingual direction [32].

Also, age could play a role in buccal bone thickness as Zhang et al. found that the apical bone width of people older than forty years is greater than that of people younger than thirty years old with a high percentage of fenestration in the young group [35].

According to this study, the cortical bone density of the mandible is higher than that of the maxilla. Other researchers observed the same results, recording an increase in the thickness and height of alveolar bone in the mandible than in the maxilla, as well as for cortical bone density [26, 36].

Several implant studies [22, 37] have found that the density of available bone is one of the factors that influence the primary stability of implants during placement. This information is valuable for surgeons operating in our community, considering modifying drilling procedures or implant choices.

Current study results suggest that bone density is generally lower in the Iraqi population than in other populations based on CBCT images, which is in agreement with Almasoud et al.'s [16] findings who asserted that the greatest bone density was at the mandibular buccal cortex of incisors (937.56 ± 176.92 HU) during their study regarding the Saudi population, in which their result of bone densities was lower than that for the normal value of densities for other populations and they attributed this to the high occurrence of “osteoporosis” in the Saudi community. However, their explanation can also be used to explain our results as Iraqi and Saudi populations share the same ethnicity and have relatively similar lifestyles and social and dietary habits.

The ovoid arch form was the dominant arch form in the current study which is in agreement with Saeed and Mageet [38] in the Sudanese population. In the same direction, Aljayousi et al. found similar results in the Jordanian population, but their second dominant arch form was the tapered (36.2%), followed by the square arch form (16.8%) [39].

The association of arch form and FCT results of the current study were in accordance with Somvasoontra et al.'s results, as they also found that the tapering arch form has the highest thickness among the different arch forms. However, their thickness was slightly higher than our measurements, but this can be attributed to the fact that they measure the whole facial bone (cortical and cancellous), while in our study, we measured only the facial cortical bone. Also, their point of measurement (level in the arch) could be higher in the bone (nearer to the base of the arch) as their point was at the root apex, while in this study, the last point was at a distance of 9 mm from the cementoenamel junction [14].

Despite there being no significance in FCT between different arch forms, care should be taken to avoid fenestration when placing an immediate implant in the ovoid arch form, as it has the lowest measurement of FCT. Thin facial cortical bone is associated with fenestration during immediate implant placement [40].

It can be suggested that preoperative evaluation of the arch form clinically will be helpful to give a preliminary clue regarding the expected treatment plan options and complications that may be faced during implant surgery to be followed by the specific investigations required to decide the final treatment plan.

The study had limitations related to teeth inclinations (labiolingual) and age. Furthermore, the assessment of cortical bone was limited to the buccal side for each incisor. Additional measurements that assessed the palatal cortical bone thickness at different levels of the root long axis with the labiolingual inclination of the tooth might have provided additional insight.

Another limitation was the small group number in some arch forms. However, considering using a different procedure for arch form evaluation that takes in the account evaluation of the position of all teeth might be more sensitive to give the exact dental arch form, whether by using a spherical system. whether by using a spherical (polar) coordinate system[41] or by utilizing computer software to obtain the “polynomial function” that best describes the curve corresponding to the dental arch form [39] could give us more detailed information regarding the expected bone thickness for each arch form. Further study that addresses these limitations is required to clarify these points and to distinguish and confirm these results.

## 5. Conclusions

The mandibular facial cortical bone width was wider than the maxillary, and the increase was upward toward the apex. The width of the facial cortical bone was less than two millimeters including both the jaws; therefore, it should have a critical evaluation before implantation in the anterior region. Bone density is higher in the mandible than in the maxilla. The ovoid arch form was the dominant arch type, and the tapering arch form had the highest alveolar bone width in the upper jaw without statistical significance.

## Figures and Tables

**Table 1:** Bone density in Hounsfield units measured at both maxilla and mandible. The densities of cortical and cancellous bones were measured 5 mm from the root apex of the coronal and interradicular regions between the two central incisors.

	*N*	Site		*P* value	Site
		Facial cortical bone	Palatal/lingual cortical bone		Cancellous bone
Maxilla	100	720.75 ± 103.17	799.46 ± 163.62	0.039	600.37 ± 126.63
Mandible	100	897.36 ± 136.72	832.86 ± 150.12	0.046	756.34 ± 143.36
*P* value		≤0.001	0.034		≤0.001

**Table 2:** Mean and standard deviation (SD) of facial cortical bone thickness and alveolar ridge width in millimeters (mm) for the upper and lower central incisors according to arch form and gender at three points of measurements ((P1: 3 mm; P2: 6 mm; and P3: 9 mm) from the cementoenamel junction). The data are presented as mean ± SD.

Arch form	Site	Facial cortical bone (FW)				Alveolar ridge (AW)			
		Upper		Lower		Upper		Lower	
		Male	Female	Male	Female	Male	Female	Male	Female
Square	P1	1.032 ± 0.087	0.992 ± 0.105	1.019 ± 0.246	1.117 ± 0.289	7.171 ± 0.392	7.308 ± 0.471	4.786 ± 0.261	4.806 ± 0.713
	P2	1.196 ± 0.071	1.253 ± 0.136	1.351 ± 0.250	1.374 ± 0.318	8.048 ± 0.240	7.800 ± 0.379	6.943 ± 0.567	5.789 ± 0.705
	P3	1.338 ± 0.297	1.413 ± 0.320	1.496 ± 0.435	1.641 ± 0.341	9.068 ± 0.485	8.467 ± 0.340	8.929 ± 0.957	6.667 ± 1.002

Ovoid	P1	1.016 ± 0.086	0.917 ± 0.255	1.072 ± 0.205	0.999 ± 0.289	7.265 ± 0.452	7.227 ± 0.425	5.013 ± 0.540	4.869 ± 0.654
	P2	1.204 ± 0.080	1.180 ± 0.168	1.378 ± 0.230	1.235 ± 0.290	8.278 ± 0.375	7.751 ± 0.359	6.944 ± 0.559	5.769 ± 0.636
	P3	1.386 ± 0.25	1.351 ± 0.330	1.644 ± 0.368	1.521 ± 0.346	8.978 ± 0.432	8.441 ± 0.358	8.665 ± 0.842	6.665 ± 0.879

Tapering	P1	1.021 ± 0.041	0.967 ± 0.053	1.097 ± 0.131	1.038 ± 0.132	7.310 ± 0.398	7.465 ± 0.344	5.170 ± 0.352	4.217 ± 0.254
	P2	1.23 ± 0.070	1.178 ± 0.111	1.41 ± 0.115	1.262 ± 0.097	8.370 ± 0.332	7.987 ± 0.307	6.770 ± 0.253	6.033 ± 0.330
	P3	1.467 ± 0.128	1.310 ± 0.209	1.785 ± 0.079	1.695 ± 0.071	9.040 ± 0.404	8.600 ± 0.379	8.530 ± 0.721	7.133 ± 0.314

## Data Availability

The data used to support the findings of this study are included within the article.
